# ADAM17 knockdown mitigates while ADAM17 overexpression aggravates cardiac fibrosis and dysfunction *via* regulating ACE2 shedding and myofibroblast transformation

**DOI:** 10.3389/fphar.2022.997916

**Published:** 2022-10-14

**Authors:** Jing Cheng, Fei Xue, Cheng Cheng, Wenhai Sui, Meng Zhang, Lei Qiao, Jing Ma, Xiaoping Ji, Wenqiang Chen, Xiao Yu, Bo Xi, Feng Xu, Guohai Su, Yuxia Zhao, Panpan Hao, Yun Zhang, Cheng Zhang

**Affiliations:** ^1^ The Key Laboratory of Cardiovascular Remodeling and Function Research, Chinese Ministry of Education, Chinese National Health Commission and Chinese Academy of Medical Sciences, The State and Shandong Province Joint Key Laboratory of Translational Cardiovascular Medicine, Department of Cardiology, Qilu Hospital, Cheeloo College of Medicine, Shandong University, Jinan, China; ^2^ Heart Center and Beijing Key Laboratory of Hypertension, Beijing Chaoyang Hospital, Capital Medical University, Beijing, China; ^3^ Key Laboratory Experimental Teratology of the Ministry of Education, Department of Physiology, School of Basic Medical Sciences, Cheeloo College of Medicine, Shandong University, Jinan, China; ^4^ Department of Epidemiology, School of Public Health, Cheeloo College of Medicine, Shandong University, Jinan, China; ^5^ Department of Emergency Medicine, Chest Pain Center, Shandong Provincial Clinical Research Center for Emergency and Critical Care Medicine, Qilu Hospital, Shandong University, Jinan, China; ^6^ Cardiovascular Disease Research Center of Shandong First Medical University, Central Hospital Affiliated to Shandong First Medical University, Jinan, China; ^7^ Department of Traditional Chinese Medicine, Qilu Hospital, Cheeloo College of Medicine, Shandong University, Jinan, China

**Keywords:** ADAM17, cardiac fibrosis, ACE2, myofibroblast transformation, TGF-β1/Smad3 signaling

## Abstract

A disintegrin and metalloprotease domain family protein 17 (ADAM17) is a new member of renin-angiotensin system (RAS) but its role in the pathogenesis of diabetic cardiomyopathy (DCM) is obscure. To test the hypothesis that ADAM17 knockdown mitigates while ADAM17 overexpression aggravates cardiac fibrosis *via* regulating ACE2 shedding and myofibroblast transformation in diabetic mice, ADAM17 gene was knocked down and overexpressed by means of adenovirus-mediated short-hairpin RNA (shRNA) and adenovirus vector carrying ADAM17 cDNA, respectively, in a mouse model of DCM. Two-dimensional and Doppler echocardiography, histopathology and immunohistochemistry were performed in all mice and *in vitro* experiments conducted in primary cardiofibroblasts. The results showed that ADAM17 knockdown ameliorated while ADAM17 overexpression worsened cardiac dysfunction and cardiac fibrosis in diabetic mice. In addition, ADAM17 knockdown increased ACE2 while reduced AT1R expression in diabetic hearts. Mechanistically, ADAM17 knockdown decreased while ADAM17 overexpression increased cardiac fibroblast-to-myofibroblast transformation through regulation of TGF-β1/Smad3 signaling pathway. In conclusion, ADAM17 knockdown attenuates while ADAM17 overexpression aggravates cardiac fibrosis *via* regulating ACE2 shedding and myofibroblast transformation through TGF-β1/Smad3 signaling pathway in diabetic mice. Targeting ADAM17 may provide a promising approach to the prevention and treatment of cardiac fibrosis in DCM.

## Introduction

The incidence of diabetes has increased rapidly in recent years, and contributed tremendously to the burden of health cost worldwide (Williams et al., 2020). Cardiovascular complications, especially diabetic cardiomyopathy (DCM), are considered as the main cause of morbidity and mortality among diabetic patients (Cai and Kang, 2003). DCM is characterized by prominent cardiac fibrosis and left ventricular remodeling and systolic and diastolic dysfunction (Nakamura and Sadoshima, 2020). Although the pathogenesis of DCM is not completely understood, the vital role of overactivated renin-angiotensin system (RAS) in the development and progression of DCM has been widely recognized and administration of angiotensin converting enzyme inhibitors (ACEIs) and angiotensin receptor blockers (ARBs) have substantially improved the clinical outcomes of patients with DCM (McDonagh and Metra, 2021). However, these classical medications have inherent limitations which make them still insufficient to counteract overactivated RAS in a large number of patients with DCM and novel approaches to counterbalance overactivated RAS in these patients are highly warranted.

A disintegrin and metalloprotease domain family protein 17 (ADAM17), also known as tumor necrosis factor-α converting enzyme (TACE), is a metallopeptidase highly expressed in multiple tissues, such as heart, vessels and brain (Dreymueller et al., 2012). ADAM17 is an important biological regulator of cellular microenvironment by shedding more than 70 cytokines and their receptors (Lichtenthaler and Meinl, 2020) and thereby regulating inflammatory responses, oxidative stress, energy metabolism, and fatty acid metabolism (Zunke and Rose-John, 2017). Recently, it has been demonstrated that ADAM17 participates in the development of several cardiovascular diseases such as hypertensive cardiomyopathy, heart failure, and atherosclerotic heart disease (Iwata et al., 2009; Xu J. et al., 2016a; Chemaly et al., 2017). Angiotensin-converting enzyme 2 (ACE2) cleaves angiotensin II (Ang II) to produce angiotensin-(1–7) [Ang-(1–7)] and thus acts as a negative regulator of RAS (Simões and Teixeira, 2016). ACE2 overexpression and administration of exogenous Ang-(1–7) alleviated Ang II or diabetes-induced cardiac hypertrophy and fibrosis (Zhai et al., 2018; Dang et al., 2020). However, ADAM17 has been found to shed ACE2 on the cell membrane into the peripheral blood (Iwata et al., 2009), which reduces transmembrane ACE2 protein level and activity in the myocardium with a corresponding increase in soluble ACE2 level and activity in the serum. In addition, Ang II may activate ADAM17 leading to enhanced ACE2 shedding, which forms negative feedback of RAS (Patel et al., 2014a). Available evidence showed that ADAM17 expression was elevated in the myocardium of patients with dilated cardiomyopathy (Fedak et al., 2006) and ADAM17 gene silencing prevented Ang II-induced cardiac hypertrophy and fibrosis in mice (Wang et al., 2009). Our previous study found that dickkopf-3 (DKK3) overexpression adequately alleviated Ang II-induced myocardial fibrosis in mice through ADAM17/ACE2 pathway (Zhai et al., 2018). However, the role of ADAM17 in the prevention and treatment of cardiac fibrosis in DCM is still unclear.

Diabetes-induced myocardial fibrosis is characterized by interstitial and perivascular collagen deposition, which ultimately results in myocardial remodeling (Jia et al., 2018). Transformation of fibroblast to myofibroblast is a key step of cardiac fibrosis as myofibroblasts are the principal producer of extracellular matrix composition (ECM), and myofibroblasts are characterized and identified by the presence of α-smooth muscle actin (α-SMA) in these cells (He et al., 2018). Cardiac fibrosis results from collagen deposition and impaired degradation of extracellular matrix composition, and myofibroblast transformation is primarily regulated by TGF-β signaling in mice with myocardial infarction (Zhou et al., 2019). However, the mechanism underlying the role of ADAM17 in myofibroblast transformation has not been clarified. Thus, the present study was undertaken to test the hypothesis that ADAM17 knockdown mitigates while ADAM17 overexpression aggravates cardiac fibrosis *via* regulating ACE2 shedding and myofibroblast transformation through TGF-β1/Smad3 signaling pathway in diabetic mice.

## Methods

### Animal model

**FIGURE 1:**
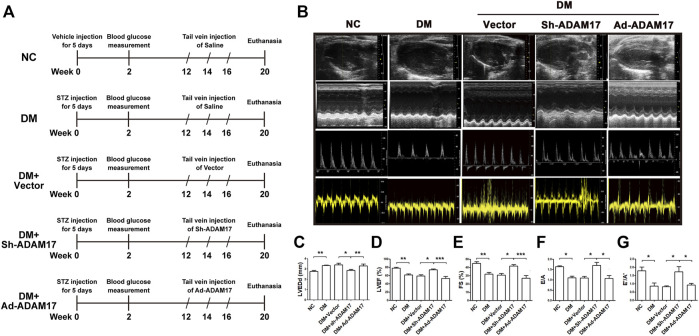
Time line of experimental studies and echocardiographic measurements in 5 groups of mice. **(A)** Animal grouping and time line of experimental studies in 5 groups of mice. **(B)** Representative two-dimensional echocardiograms (first row), M-mode echocardiograms (second row), pulse-wave Doppler spectra of mitral inflow (third row), and tissue Doppler spectra of mitral annulus (fourth row). **(C)** Measurements of left ventricular end-diastolic diameter (LVEDd) in 5 groups of mice. **(D)** Measurements of left ventricular ejection fraction (LVEF) in 5 groups of mice. **(E)** Measurements of fractional shortening (FS) in 5 groups of mice. **(F)** Measurements of ratio of early to late left ventricular filling velocity (E/A) in 5 groups of mice. **(G)** Measurements of ratio of early to late diastolic peak annular velocity (E’/A′) in 5 groups of mice. Data were expressed as the means ± SEM. *p < 0.05, **p < 0.01, ***p < 0.001. *n* = 10 per group.

Fifty C57BL/6J male mice aged 8 weeks were randomly assigned to five groups: normal control (NC) group, diabetes mellitus (DM) group, DM + adenovirus vector (DM + Vector) group, DM + ADAM17-shRNA group, and DM + Ad-ADAM17 group. All mice except for NC group received intraperitoneal injection of streptozotocin (STZ, Sigma-Aldrich, St Louis, MO) dissolved in 0.2 ml vehicle (0.1 M citrate buffer; pH 4.5) at a dosage of 55 mg/kg for five consecutive days to induce type 1 DM. Mice in the NC group received intraperitoneal injection of equivalent volume of saline. In all mice, fasting blood glucose levels were measured by a glucometer (ACCU-CHEK Advantage; Roche, Indianapolis, IN) 1 week after STZ injection. Only mice with blood glucose levels ≥16.7 mM were included in the four DM groups. Adenovirus-mediated shRNA against murine ADAM17 (ADAM17 shRNA) or adenovirus-mediated ADAM17 cDNA (Ad-ADAM17) was used to knockdown or increase ADAM17 expression, respectively (Figure 1A, Protocol). The sequence of ADAM17 shRNA was 5′-GGA​CCA​AGG​AGG​AAA​GTA​T-3′. At 12, 14 and 16 weeks after diabetes induction, adenovirus vector carrying ADAM17 cDNA (SinoGenoMax, Beijing, China), 5×10^9^ UT/200 uL adenovirus vector carrying ADAM17 shRNA (Genechem, Shanghai, China), and adenovirus vector of equivalent volume (Genechem, Shanghai, China) were injected *via* the tail vein in the DM + adenovirus vector group, DM + ADAM17-shRNA group and DM + Ad-ADAM17 group, respectively. All mice were fed with a normal diet throughout the experiment and body weight was measured before euthanasia. All animal experiments were in accordance with the Guide of the Care and Use of Laboratory Animals (NIH Publication No. 86–23, revised 1996) and approved by the Animal Care and Use Committee of Shandong University Qilu Hospital (Jinan, China).

### Two-dimensional and Doppler echocardiography

Left ventricular systolic and diastolic function was assessed by transthoracic echocardiography using Vevo2100 imaging system (VisualSonics, Toronto, Canada) in all mice after anesthesia with isoflurane. Left ventricular end-diastolic diameter (LVEDd) and left ventricular ejection fraction (LVEF) and fractional shortening (FS) were measured by M-mode echocardiography in the left ventricular long axis view. The early (E) and late (A) diastolic mitral flow velocities were measure by pulsed Doppler in the four-chamber view and the ratio of E/A was calculated. The early (E′) and late (A′) diastolic mitral annular velocities were measure by tissue Doppler imaging in the four-chamber view and the ratio of E’/A’ was derived.

### Histopathology and immunohistochemistry

Mouse hearts were bisected transversely at the mid-ventricular level and then fixed in 4% paraformaldehyde for 12h. Paraffin-embedded specimens were sectioned (4 μm thick of slide) for subsequent experiments. Masson’s trichrome staining was performed to detect myocardial fibrosis. Sections were incubated with primary antibody of anti-ACE2 (dilution, 1:100; Abcam, Cambridge, MA) overnight at 4°C and subsequently incubated with goat anti-rabbit secondary antibody for 30 min at 37°C. Sections were stained with diaminobenzidine and counterstained with hematoxylin. Sections were analyzed using Image-Pro Plus 6.0.

### Cell culture

Primary cardiofibroblasts were isolated from 1- to 3-day-old mouse as described previously (Kapadia et al., 2000). Briefly, hearts were excised from 1- to 3-day-old mouse, minced, and digested in 0.08% trypsin (GIBCO) in calcium-free Hanks’ balanced salt solution (DHEPES) by serial digestion. Cardiofibroblasts were separated after 2-hr adhesion and cultured in Dulbecco’s modified Eagle’s medium containing 10% fetal bovine serum and 2 mM glutamine at 37°C under 5% CO_2_ and 95% relative humidity condition. Cardiofibroblasts at 60% confluence were randomly divided into 6 groups who were disposed to different treatment: 1) 5.5 mM glucose (low glucose control, NC); 2) combination of 5.5 mM glucose and 27.8 mM mannitol (high osmotic pressure control, HO); 3) 33.3 mM glucose (high glucose, HG); 4) adenovirus-mediated scrambled shRNA prior to high glucose treatment (high glucose and scrambled vector (HG + Vector); 5) adenovirus-mediated ADAM17 shRNA prior to high glucose treatment (high glucose and ADAM17-ShRNA, HG + Sh-ADAM17) and 6) adenovirus-mediated ADAM17 plasmid prior to high glucose treatment (high glucose and ADAM17 plasmid, HG + Ad-ADAM17). To examine the effects of ADAM17 on fibroblast-myofibroblast transition, scrambled vector, adenovirus-mediated ADAM17 shRNA and ADAM17 plasmid were transfected 24 h prior to stimulation with different concentrations of glucose in cardiofibroblasts.

### Quantitative real-time RT-PCR

Total RNA was extracted from primary cardiofibroblasts by using RNA Extraction Kit (CWBIO, Beijing, China). Applied Biosystems cDNA Reverse Transcription and CWBIO SYBR RT-PCR kits were used for quantitative real-time RT-PCR. The primer sequences for ADAM17, ACE2 and β-actin genes are listed in Supplementary Table S1. Quantitative values were obtained from the threshold cycle value (Ct value) and relative mRNA expression levels were analyzed by the 2^-△△Ct^ method.

### ADAM17 activity assay

After treatment, cardiofibroblasts were lysed by activity assay buffer (pH 7.4; 50 mM Tris HCl, 25 mM NaCl, 4% glycerol and 10 mM ZnCl_2_). ADAM17 activity was measured in triplicate for each sample by using the SensoLyte 520 TACE (α-Secretase) Activity Assay Kit (AnaSpec, Fremont, CA).

### Enzyme-linked immunosorbent assay

Supernatant concentration of ACE2 was measured by ELISA kit (OmnimAbs, CA) following the manufacturer’s instructions. All samples were detected in triplicate in a blinded manner.

### Immunofluorescence staining

After treatment, cardiofibroblasts were washed with PBS and fixed with 4% paraformaldehyde for 15 min at room temperature. Then cells were blocked by 5% donkey serum (Sigma-Aldrich, St Louis, MO) for 1 h at room temperature after rinsing thoroughly. Cardiofibroblasts were incubated overnight at 4°C with anti-S100A4 (1:100; Proteintech, Wuhan, China) and anti-α-SMA (1:200; Abcam). Sections were then incubated with fluorescent secondary antibodies conjugated with Alexa Fluor 594-conjugated anti-rabbit (1:400; Abcam) and Alexa Fluor 488 anti-mouse (1:400; Abcam) for 1 h at room temperature. Nuclei were counterstained with 4’,6 diamino-2-phenylindole (DAPI, Abcam). Images were captured though laser scanning confocal microscopy (ECLIPSE-Ni, Nikon, Japan).

### Western blot analysis

Proteins from cardiofibroblasts and murine heart tissues were separated by 10% sodium dodecyl sulfate-polyacrylamide gel electrophoresis (SDS-PAGE). Then proteins were transferred to PVDF membrane from SDS-PAGE gel. Proteins in PVDF membrane were incubated with primary antibodies for collagen I (1:1,000; Abcam), collagen III (1:1,000; Abcam), TGF-β1 (1:1,000; Abcam), ADAM17 (1:1,000; Abcam), ACE2 (1:1,000; Abcam), Ang receptor type I (AT1R) (1:1,000; Abcam), Ang receptor type II (AT2R) (1:1,000; Abcam), MasR (1:1,000; OmnimAbs, CA), α-SMA (1:1,000; Abcam), p-p38 (1:1,000; Cell Signaling Technology, Danvers, MA), p38 (1:1,000; Cell Signaling Technology), p-Smad3 (1:1,000; Cell Signaling Technology), Smad3 (1:1,000; Cell Signaling Technology), and GAPDH (1:1,000; Abcam) overnight at 4°C. The membranes were then washed and incubated with horseradish peroxidase-conjugated secondary antibodies (1:5,000; Proteintech, Wuhan, China) for 1 h at room temperature. Protein levels of collagen I, collagen III, ADAM17, ACE2 and TGF-β1 were normalized to that of GAPDH, and levels of phospho-proteins were normalized to that of total proteins.

### Data analysis

Continuous data were reported as the mean ± standard error of mean (SEM). Comparisons were analyzed by one-way analysis of variance (ANOVA) and Post hoc Bonferroni tests were performed in case of significant interactions of ANOVAs. *p* value <0.05 was considered statistically significant. GraphPad Prism version 7.0 (GraphPad Software Inc., San Diego, CA) was used for statistical analysis.

## Results

### ADAM17 knockdown ameliorated while ADAM17 overexpression worsened cardiac dysfunction in diabetic mice

At the end of the experiment, blood glucose level was significantly increased in all diabetic groups compared with the normal control group. However, blood glucose level did not differ between DM + adenovirus vector group and DM + ADAM17-shRNA group or DM + Ad-ADAM17 group (Supplementary Figure S1A).

Echocardiography was used to assess left ventricular systolic and diastolic function in all mice before euthanasia. As shown in Figure 1, LVEDd was increased whereas LVEF, FS, E/A and E’/A′ were decreased in DM and DM + Vector groups of mice in comparison with the NC group (Figures 1B–G). However, these measurements in cardiac size and function were substantially improved in the ADAM17-shRNA group relative to the DM + Vector group and basically returned to the NC group levels (Figures 1B–G). In contrast, LVEDd was again increased whereas LVEF, FS, E/A and E’/A’ were decreased in DM + Ad-ADAM17 group in comparison with NC group and these differences were more significant for values of LVEF and FS. These results indicated that ADAM17 knockdown ameliorated while ADAM17 overexpression worsened cardiac dysfunction in diabetic mice.

### ADAM17 knockdown prevented while ADAM17 overexpression promoted cardiac fibrosis in diabetic mice

**FIGURE 2:**
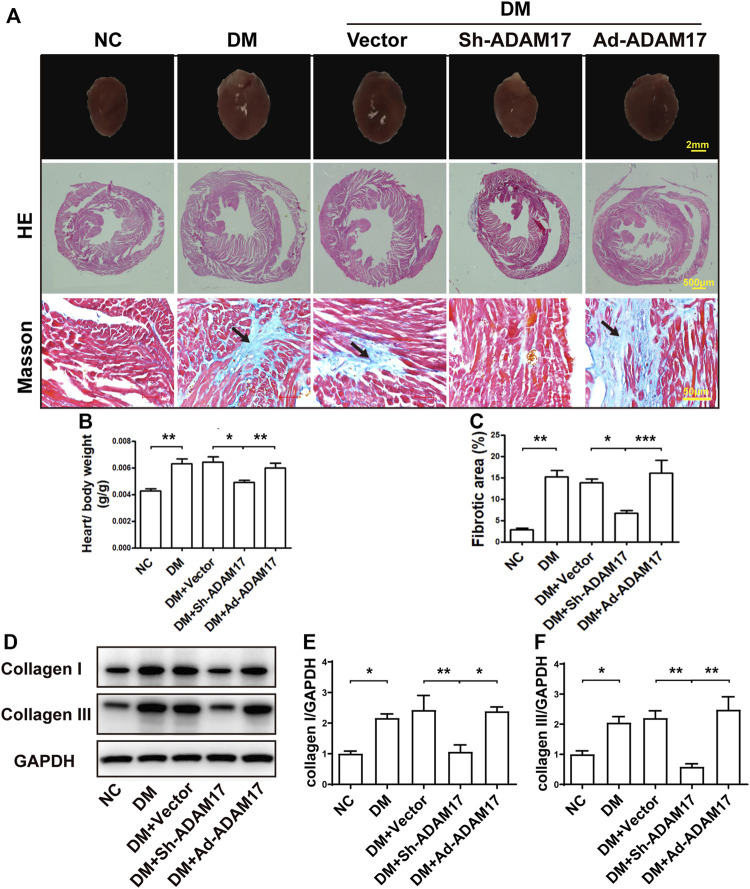
Comparison of heart size, cardiac fibrosis and myocardial collagen deposition in 5 groups of mice. **(A)** Representative images of cardiac cross sections (first row; scale bar: 2 mm), hematoxylin and eosin (HE) staining (second row, 40x, scale bar: 500 μm; third row, 400x, scale bar: 50 μm) and Masson’s trichrome staining (fourth row; 400x, scale bar: 50 μm) in 5 groups of mice. **(B)** Measurements of heart/body weight ratio in 5 groups of mice. **(C)** Measurements of fibrotic area in cardiac cross sections in 5 groups of mice. **(D)** Representative Western blot images and quantification of collagen I **(E)** and III **(F)** expression in 5 groups of mice. Data were expressed as the means ± SEM. *p < 0.05, **p < 0.01, ***p < 0.001. *n* = 6 per group.

HE staining showed that the heart size and the left ventricular cross-sectional area were larger in DM and DM + Vector groups than in the NC group, and normalized in the ADAM17-shRNA group, which were again increased in Ad-ADAM17 group (Figure 2A). Heart-to-body weight ratio was increased in DM and DM + Vector groups of mice *versus* the NC group, which was largely reversed in the DM + ADAM17-shRNA group but resumed in the DM + Ad-ADAM17 group (Figure 2B). Masson’s trichrome staining showed that the left ventricular fibrotic area was dramatically increased in DM and DM + Vector groups of mice compared with that in the NC group, and such an increase was substantially abolished by ADAM17-shRNA treatment while reverted to a higher level by Ad-ADAM17 treatment relative to the DM + Vector group (Figures 2A,C). Furthermore, mice in the DM and DM + Vector groups exhibited a higher level of collagen I and collagen III expression compared with the NC group, which was offset by ADAM17-shRNA treatment but resumed by Ad-ADAM17 treatment (Figures 2D–F). These results suggested that ADAM17 inhibition prevented while ADAM17 overexpression promoted cardiac fibrosis in diabetic mice.

### ADAM17 knockdown increased ACE2 while reduced AT1R expression in diabetic hearts

**FIGURE 3:**
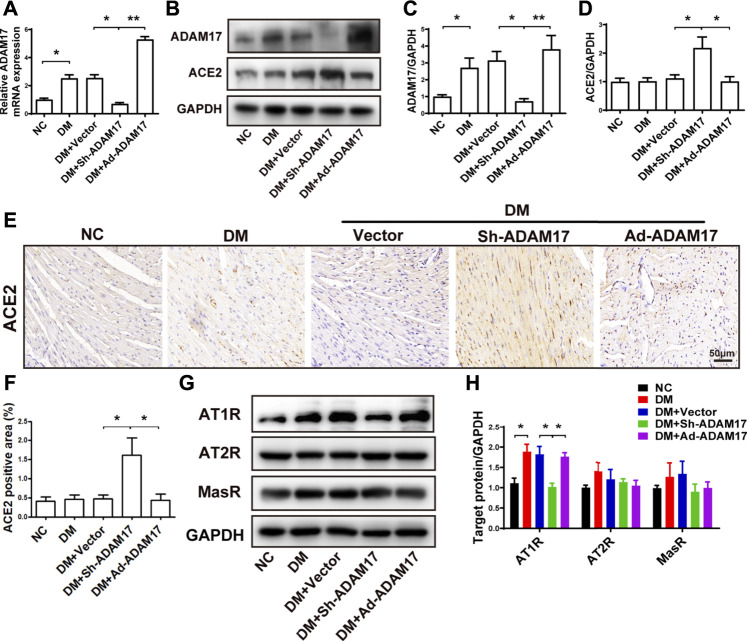
Comparison of ADAM17, ACE2, AT1R, AT2R and MasR expression in 5 groups of mice. **(A)** Quantification of mRNA level of ADAM17 in 5 groups of mice. **(B)** Representative Western blot images of ADAM17 and ACE2 in 5 groups of mice**. (C)** Quantification of protein expression of ADAM17 in 5 groups of mice. **(D)** Quantification of protein expression of ACE2 in 5 groups of mice. **(E)** Representative immunohistochemical staining of ACE2 (Scale: 50 μm) in 5 groups of mice. **(F)** Quantitative analysis of ACE2 expression in 5 groups of mice. **(G)** Representative Western blot images of AT1R, AT2R and MasR in 5 groups of mice. **(H)** Quantification of protein expression of AT1R, AT2R and MasR in 5 groups of mice. Data were expressed as the means ± SEM. *p < 0.05, **p < 0.01, ***p < 0.001. *n* = 6 per group.

In comparison with the NC group, ADAM17 mRNA and protein expression in the myocardium was significantly increased in the DM group relative to the NC group, possibly due to an activated RAS in the setting of diabetes. However, ADAM17-shRNA treatment reduced cardiac ADAM17 mRNA and protein levels virtually to the levels of NC group which were again raised to high levels by Ad-ADAM17 treatment (Figures 3A–C). These results verified the successful knockdown and overexpression of ADAM17 in our DCM model, which were the premise of imaging, morphological, and molecular biological changes in these animals. By comparison, ACE2 expression did not differ between NC and DM or DM + Vector groups. However, ADAM17-shRNA treatment significantly increased the expression level of ACE2 protein in diabetic hearts as detected by both Western blot (Figures 3B,D) and immunohistochemistry (Figures 3E,F). In addition, AT1R protein expression was markedly higher in DM or DM + Vector groups than the NC group, possibly due to activated RAS and increased Ang II secretion in diabetic hearts ae revealed by our previous studies (Dong et al., 2012). ADAM17-shRNA treatment significantly reduced AT1R protein level as compared with DM + Vector group (Figures 3G,H), which was reverted by Ad-ADAM17 treatment. In contrast, no difference was observed in AT2R and MasR protein levels among five groups of mice.

### ADAM17 knockdown decreased while ADAM17 overexpression increased myofibroblast transformation in diabetic hearts

**FIGURE 4:**
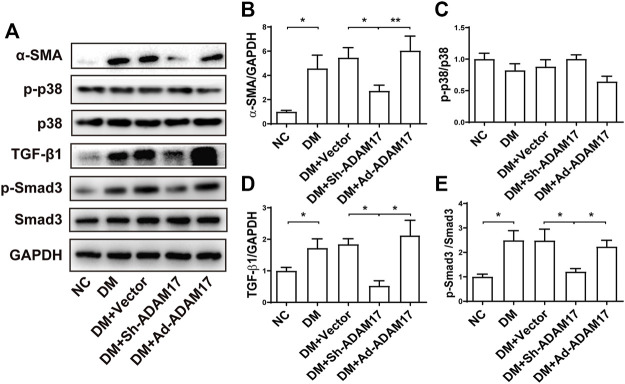
Comparison of α-SMA, p38, TGF-β1, and Smad3 expression in 5 groups of mice. **(A)** Representative Western blot images of α-SMA, p-p38, p38, TGF-β1, p-Smad3 and Smad3 in 5 groups of mice. **(B)** Quantification of α-SMA protein expression in 5 groups of mice**. (C)** Quantification of p38 phosphorylation in 5 groups of mice. **(D)** Quantification of TGF-β1 protein expression in 5 groups of mice. **(E)** Quantification of Smad3 phosphorylation in 5 groups of mice. Data were expressed as the means ± SEM. *p < 0.05, **p < 0.01, ***p < 0.001. *n* = 6 per group.

As shown in Figure 4, the protein expression of α-SMA, a unique marker of cardio-myofibroblast, was remarkably increased in DM or DM + Vector groups relative to the NC group, which was blunted by ADAM17-shRNA treatment while raised again to a even higher level by Ad-ADAM17 treatment (Figures 4A,B). There was no significant difference in p38 phosphorylation level among 5 groups of mice (Figures 4A,C). Both TGF-β1 protein expression and Smad3 phosphorylation levels were significantly higher in DM or DM + Vector groups than the NC group, which were reduced to the NC group level in the ADAM17-shRNA group and resumed in the Ad-ADAM17 group (Figures 4A,D,E). Taken together, ADAM17 knockdown decreased while ADAM17 overexpression increased myofibroblast transformation in diabetic hearts.

### Effects of high glucose treatment and ADAM17 gene manipulation on RAS *in vitro*

**FIGURE 5:**
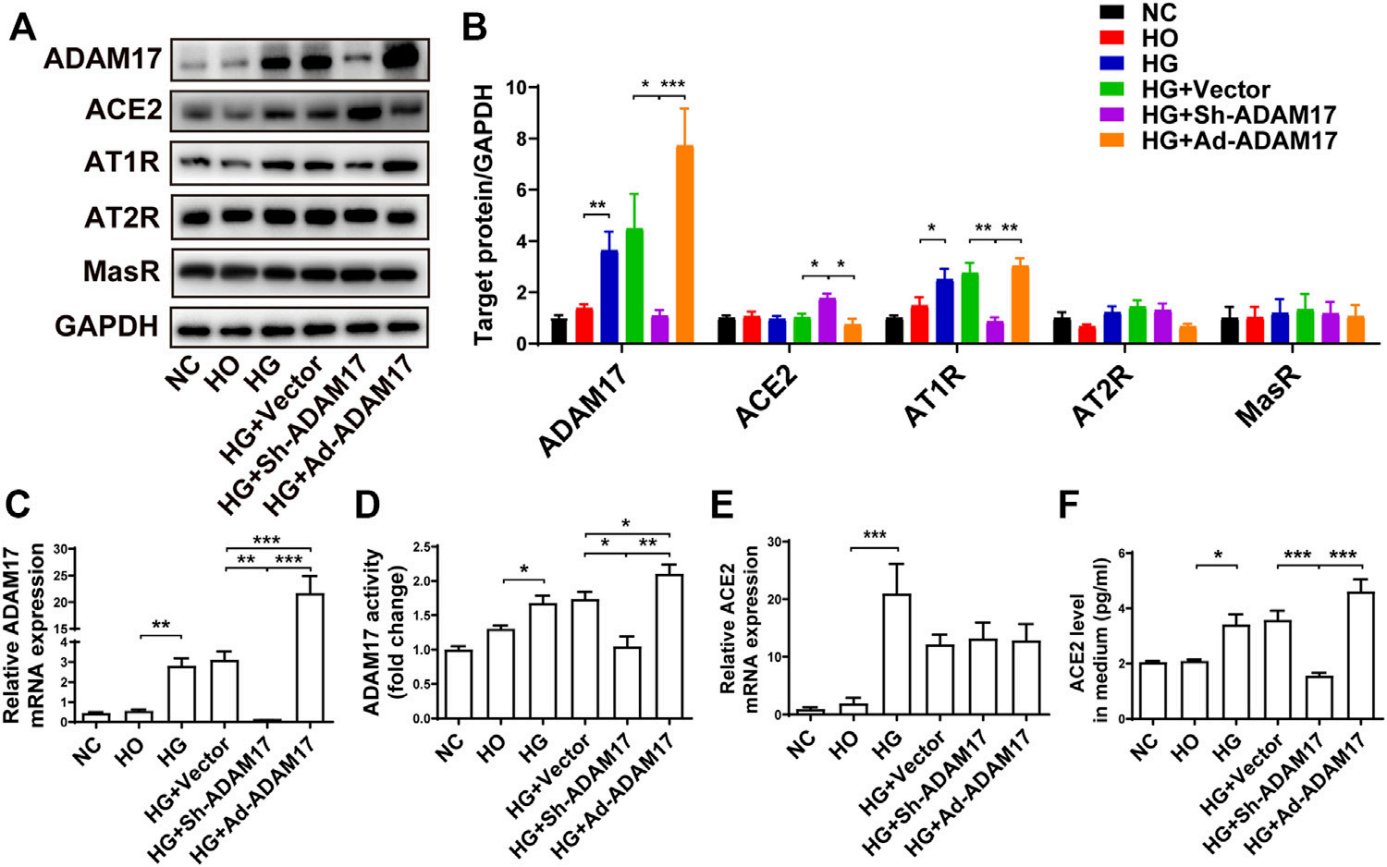
Comparison of ADAM17, ACE2, AT1R, AT2R and MasR expression in 6 groups of primary cardiofibroblasts. **(A)** Representative Western blot images of ADAM17, ACE2, AT1R, AT2R, and MasR in 6 groups of cardiofibroblasts. **(B)** Quantification of protein expression of ADAM17, ACE2, AT1R, AT2R, and MasR in 6 groups of cardiofibroblasts. **(C)** Quantification of mRNA level of ADAM17 in 6 groups of cardiofibroblasts. **(D)** Quantification of ADAM17 activity in 6 groups of cardiofibroblasts. **(E)** Quantification of mRNA level of ACE2 in 6 groups of cardiofibroblasts. **(F)** Quantification of ACE2 expression in the medium supernatant of 6 groups of cardiofibroblasts. Data were expressed as the means ± SEM. *p < 0.05, **p < 0.01, ***p < 0.001. *n* = 6 per group.

Primary cardiofibroblasts were divided into 6 groups depending to different treatments (NC, HO, HG, HG + Vector, HG + Sh-ADAM17, and HG + Ad-ADAM17 groups). The HO group was used to assess the effect of a higher osmotic pressure on protein expression in the four HG groups *versus* the NC group. As no significant difference in the protein expression of ADAM17, ACE2, AT1R, AT2R and MasR was noted between the NC and HO groups, the possibility that a high osmotic pressure in cell culture solution may alter protein expression in the HG groups can be eliminated. (Figures 5A,B). ADAM17 mRNA and protein expression was significantly increased in the HG and HG + Vector groups relative to the NC groups, which returned to the baseline level in the HG + Sh-ADAM17 group while raised again in the HG + Ad-ADAM17 group (Figures 5A–D). ACE2 mRNA level was dramatically increased in cardiofibroblasts exposed to high glucose compared with the NC group, which was not altered by ADAM17 knockdown or overexpression (Figure 5E). No difference in ACE2 protein level was observed among cardiofibroblasts in the NC, HO, HG and HG + Vector, which was similar to our *in vivo* finding that ACE2 protein expression did not differ between NC and DM or DM + Vector groups of mice. In contrast, ADAM17 knockdown significantly increased ACE2 protein level in cardiofibroblasts stimulated with high glucose, which was abolished by ADAM17 overexpression (Figures 5A,B). By comparison, ACE2 protein level in the cell supernatant was significantly increased in HG group *versus* the NC and HO groups, and ADAM17 shRNA treatment significantly reduced ACE2 level in the supernatant (Figure 5F), indicating that ADAM17 may cleave ACE2 into supernatant under high glucose conditions. Furthermore, ADAM17 knockdown significantly reduced AT1R protein expression in cardiofibroblasts cultured in high glucose as compared with scramble vector treatment (Figures 5A,B). However, there was no significant difference in AT2R and MasR protein expressions in cardiofibroblasts among 6 groups of cardiofibroblasts (Figures 5A,B).

### ADAM17 knockdown and overexpression affected myofibroblast transformation through regulation of TGF-β1/Smad3 pathway *in vitro*

**FIGURE 6:**
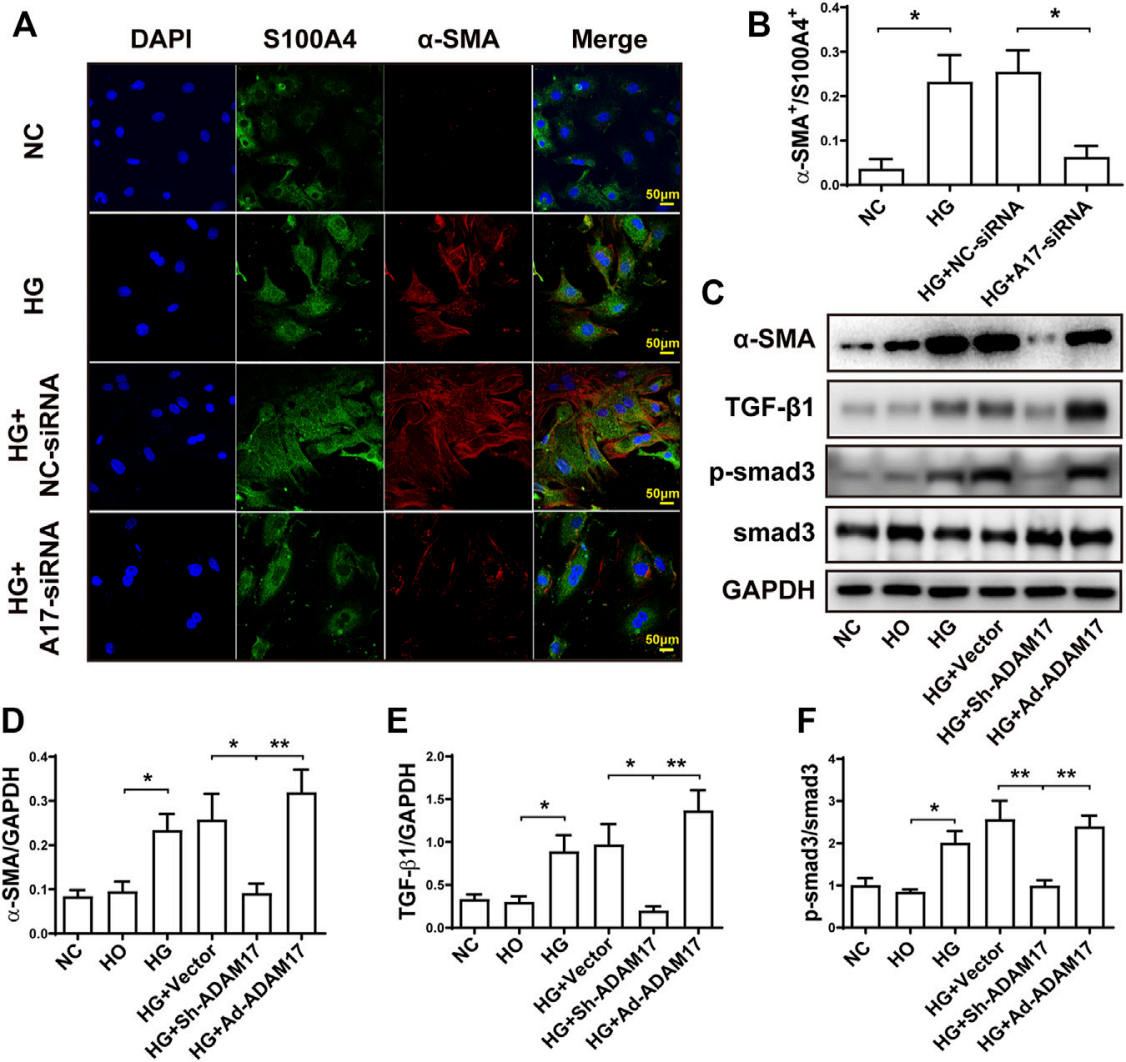
Comparison of α-SMA, S100A4, TGF-β1 and Smad3 in primary cardiofibroblasts. **(A)** Representative immunohistochemical staining of S100A4 and α-SMA (400x, scale bar: 50 μm) in 4 groups of cardiofibroblasts. **(B)** Quantification of α-SMA^+^/S100A4^+^ ratio in 4 groups of cardiofibroblasts. **(C)** Representative Western blot images of α-SMA, TGF-β1, Smad3 phosphorylation and Smad3 in 6 groups of cardiofibroblasts. **(D)** Quantification of α-SMA protein expression in 6 groups of cardiofibroblasts. **(E)** Quantification of TGF-β1 protein expression in 6 groups of cardiofibroblasts. **(F)** Quantification of Smad3 phosphorylation level in 6 groups of cardiofibroblasts. Data were expressed as the means ± SEM. *p < 0.05, **p < 0.01, ***p < 0.001. *n* = 6 per group.

We further examined the effect of ADAM17 knockdown and overexpression on myofibroblast transformation and the underlying mechanism in primary cardiofibroblasts. As shown in Figure 6, the green and red staining represented S100A4-positive and α-SMA-positive cells, respectively, where S100A4 is a marker of cardiofibroblasts and α-SMA is a marker of myofibroblasts. High glucose stimulation significantly increased α-SMA^+^/S100A4^+^ ratio compared with NC group, while this ratio was normalized in the HG + ADAM17-siRNA group (Figures 6A,B). In addition, high glucose treatment significantly increased α-SMA and TGF-β1 protein expressions as well as Smad3 phosphorylation in cardiofibroblasts, relative to the NC group, which were reversed by ADAM17 knockdown but increased again by ADAM 17 overexpression (Figures 6C–F).

## Discussion

There were several important findings in the present study. Frist, ADAM17 knockdown ameliorated while ADAM17 overexpression worsened cardiac dysfunction and cardiac fibrosis in diabetic mice. Second, ADAM17 knockdown increased ACE2 while reduced AT1R expression in diabetic hearts; Third, ADAM17 knockdown decreased while ADAM17 overexpression increased myofibroblast transformation through regulation of TGF-β1/Smad3 pathway. To the best of our knowledge, our study is the first to demonstrate the opposite effects of ADAM17 knockdown and overexpression on cardiac fibrosis in diabetic hearts through regulating ACE2 shedding and myofibroblast transformation.

Ample evidence indicates that overactivation of RAS plays a key role in the pathogenesis of cardiac fibrosis in diabetic cardiomyopathy and attempts at diminishing RAS activity ameliorate cardiac pathology and function in this condition. ACE2, a homolog of ACE, is expressed widely in the heart which converts octapeptide angiotensin Ang II to heptapeptide Ang-(1–7), thus mitigating the deleterious effects of RAS (Nunes-Silva et al., 2017). Recent studies found that ACE2 ectodomain can be cleaved by ADAM17 and then released from the cell membrane into the extracellular milieu (Xu et al., 2017)**.** In the present study, ACE2 mRNA level was increased in high glucose-treated cardiofibroblasts relative to the NC group, whereas ACE2 protein levels were not increased in HG-treated cardiofibroblasts or diabetic mice relative to the normal control group, which might be because an upregulated ACE2 mRNA level may increase ACE2 protein expression whereas upregulated ADAM17 expression may enhance ACE2 shedding in the diabetic condition, resulting in an unchanged ACE2 protein expression. However, ADAM17 knockdown significantly increased ACE2 protein levels in both diabetic hearts and HG-treated cardiofibroblasts. Similarly, protein levels of ADAM17 and AT1R were increased in diabetic hearts and HG-treated cardiofibroblasts, which were both reversed by ADAM17 knockdown. Patel et al. also found that Ang II infusion upregulated the expression of myocardial ADAM17, which was mediated by AT1R, and ADAM17 inhibition prevented ACE2 shedding (Patel et al., 2014a). Thus, our study provided evidence that ADAM17 knockdown diminished ACE2 shedding, thus preserving the ACE2 function to convert profibrotic Ang II to antifibrotic Ang-(1–7). As HG stimulation upregulated ADAM17 protein expression in cardiofibroblasts, ACE2 level in supernatant medium was increased secondary to enhanced ACE2 shedding. In contrast, ADAM17 knockdown significantly decreased ACE2 shedding, resulting in a declined ACE2 level in supernatant medium. Nonetheless, either knockdown or overexpression of ADAM17 exerted no effect on the mRNA level of ACE2.

DCM, as a serious diabetic complication, is characterized as abnormal cardiac structure and function. Cardiac fibrosis is a salient pathological feature of DCM manifested as accumulation of extracellular matrix proteins including collagen I and III, which may lead to an increased left and right ventricular stiffness and cardiac diastolic dysfunction (Hao P. P. et al., 2015b). Progressive cardiac fibrosis may ultimately induce cardiac systolic dysfunction and overt heart failure (Evangelista et al., 2019). Numerous studies have shown that TGF-β1 is an important mediator in fibrogenesis and DCM progression (Yue et al., 2017). In the current study, two-dimensional and Doppler echocardiography revealed left ventricular systolic and diastolic dysfunction in diabetic mice, which was significantly improved by ADAM17 shRNA treatment. In addition, ADAM17 knockdown decreased collagen deposition and downregulated collagen I, collagen III and TGF-β1 expression in diabetic hearts. On the contrary, ADAM17 overexpression enhanced collagen deposition and expression in the myocardium of diabetic mice. These results lend support to previous observations that ADAM17 systemic knockdown prevented cardiac hypertrophy and myocardial fibrosis in spontaneously hypertensive rodents (Wang et al., 2009), and ADAM17 antibody prevented cardiac hypertrophy and fibrosis induced by Ang II infusion (Takayanagi et al., 2016). Thus, ADAM17 inhibition reduced collagen I, collagen III and TGF-β1 expression, and ameliorated cardiac fibrosis in diabetic cardiomyopathy.

TGF-β, as a master regulator in tissue fibrosis, consists of three isoforms, TGF-β1, TGF-β2 and TGF-β3 (Zhao et al., 2020). TGF-β activation promoted myofibroblast differentiation and secretion of ECM components, such as collagen and fibronectin (Wang et al., 2021). TGF-β1 mediated fibrosis *via* its downstream Smad3 rather than Smad2 signaling (Meng et al., 2010). Loss of Smad3 reduced cardiac fibrosis and improved cardiac compliance (Biernacka et al., 2015). Besides, Smad3 binds to the promoter of α-SMA in mouse cardiofibroblasts and regulates its transcription (Li et al., 2011). Deletion of Smad3 protected against cardiac fibrosis under a number of conditions such as hypertension, myocardial infarction, and diabetes (Li et al., 2011). In addition, Smad3-deficient mice demonstrated reduction in both renal and cardiac fibrosis in STZ-induced type 1 diabetes and in obese diabetic mice (Wang et al., 2007; Biernacka et al., 2015). Taken together, accumulating evidence suggests that TGF-β/Smad3 signaling plays an important role in cardiovascular diseases, especially in the pathogenesis of DCM (Wang et al., 2021).

Upon myocardial injury, resting cardiac fibroblasts are transdifferentiated into contractile and secretory myofibroblasts, which produce and secret a large amount of TGF-β1, collagen I and collagen III to promote cardiac repairment. However, sustained activation of myofibroblasts may lead to excessive accumulation of extracellular matrix and cardiac fibrosis (Weber et al., 2013). As the quantity of cardiac fibroblasts are overwhelmingly beyond that of cardiomyocytes, an increased fibroblast-to-myofibroblast transformation have been recognized as an important biomarker of cardiac fibrosis (Frangogiannis, 2019). In this study, the expression α-SMA, a marker of myofibroblasts, was significantly increased in high glucose-treated cardiofibroblasts, indicating an enhanced fibroblast-to-myofibroblast transformation, which was reversed by ADAM17 knockdown, suggesting a normalized fibroblast-myofibroblast transformation. In addition, previous studies have shown that both canonical and non-canonical TGF-β signaling pathways play an important role in the development of cardiac fibrosis (Finnson et al., 2020). In light of the interplay between ADAM17 and cardiac fibrosis, there may be a possible link between ADAM17 and TGF-β. In the present study, we found that ADAM17-shRNA treatment reversed the elevated levels of TGF-β1 protein expression and decreased the phosphorylation level of Smad3 in diabetic mice. This is consistent with a previous study where 25-O-methylalisol F (MAF), a novel RAS inhibitor, was found to attenuate tubulo-interstitial fibrosis and epithelial-mesenchymal transition *via* inhibiting Smad3 signaling without altering Smad2, PI3K, ERK1/2 and p38 phosphorylation in Ang II-stimulated fibroblasts (Chen et al., 2018). In addition, TGF-β was reported to promote fibroblast-to-myofibroblast transformation, and canonical TGF-β signaling upregulated Smad2/3 transcription factors thereby inducing cardiac fibrosis (Khalil et al., 2017). Thus, our results supported the notion that ADAM17 knockdown ameliorates cardiac fibrosis probably *via* TGF-β/Smad3 signaling in diabetes mice. Besides, a recent study showed that ADAM17 knockdown inhibited the expression of TGF-β in gastric carcinoma cells, with the downstream Smad2 and Smad3 also being attenuated (Xu M. et al., 2016b). Furthermore, Kawasaki disease and secondary coronary artery lesions are found to be associated with ADAM17 genetic variants through TGF-β/Smad3 signaling pathway (Peng et al., 2016). Thus, ADAM17 inhibition may play a protective role in cardiac fibrosis, which is likely mediated by TGF-β/Smad3 signaling pathway.

The majority of detrimental effects of Ang II is mediated *via* AT1R activation (Patel et al., 2014b). High glucose stimulation increases intracellular Ang II production in cardiomyocytes, resulting in an activation of the Ang II/AT1R pathway (Sukumaran et al., 2017). Previous studies demonstrated that administration of ACE2 activator increased ACE2 expression and activity while decreased AT1R expression in the myocardium of rats with DCM (Murça et al., 2012a; Murça et al., 2012b). In the present study, ADAM17 knockdown upregulated ACE2 expression and downregulated AT1R expression, but exerted no effect on AT2R and MasR expression. The study by Wang et al., who used ADAM17 knockdown to treat Ang II-induced cardiac hypertrophy and fibrosis in mice, did not explore the effect of ADAM17 knockdown on AT1R, AT2R and MasR expression (Wang et al., 2009). Other studies that used Ang-(1–7) or azilsartan to treat DCM in rats or mice found a significant reduction in AT1R expression in the myocardium but the effect on AT2R and MasR expression was variable (Hao P. et al., 2015a; Sukumaran et al., 2017). Thus, the major therapeutic benefit of ADAM17 knockdown in the current mouse model of DCM derived from AT1R inhibition.

There were a couple of limitations to the present study. First, adenovirus-mediated shRNA against murine ADAM17 was used to inhibit ADAM17 activity although cardiac fibroblast-specific knockout of ADAM17 may more effectively remove the function of ADAM17 in the pathogenesis of cardiac fibrosis. As there is no specific marker of cardiac fibroblast, we created a mouse model of fibroblast-specific knockout of ADAM17 and found severe sepsis and high mortality in these mice with unclear mechanism. To date, there has been no report in the literature of a successful mouse model of cardiac fibroblast-specific knockout of ADAM17. Second, although we found that ADAM17 knockdown ameliorated cardiac fibrosis probably *via* TGF-β/Smad3 signaling, we did not testify the causal relation between TGF-β/Smad3 signaling and cardiac fibrosis in our mouse model, and further studies are warranted in this regard. Finally, it has been shown that oxidative stress plays a major role in myocardial ischemia reperfusion injury (Chen et al., 2021; Zhao et al., 2022) and DCM (Ren et al., 2020), and it requires further investigation whether the therapeutic effects of ADAM17 knockdown on DCM involve suppression of oxidative stress.

**FIGURE 7:**
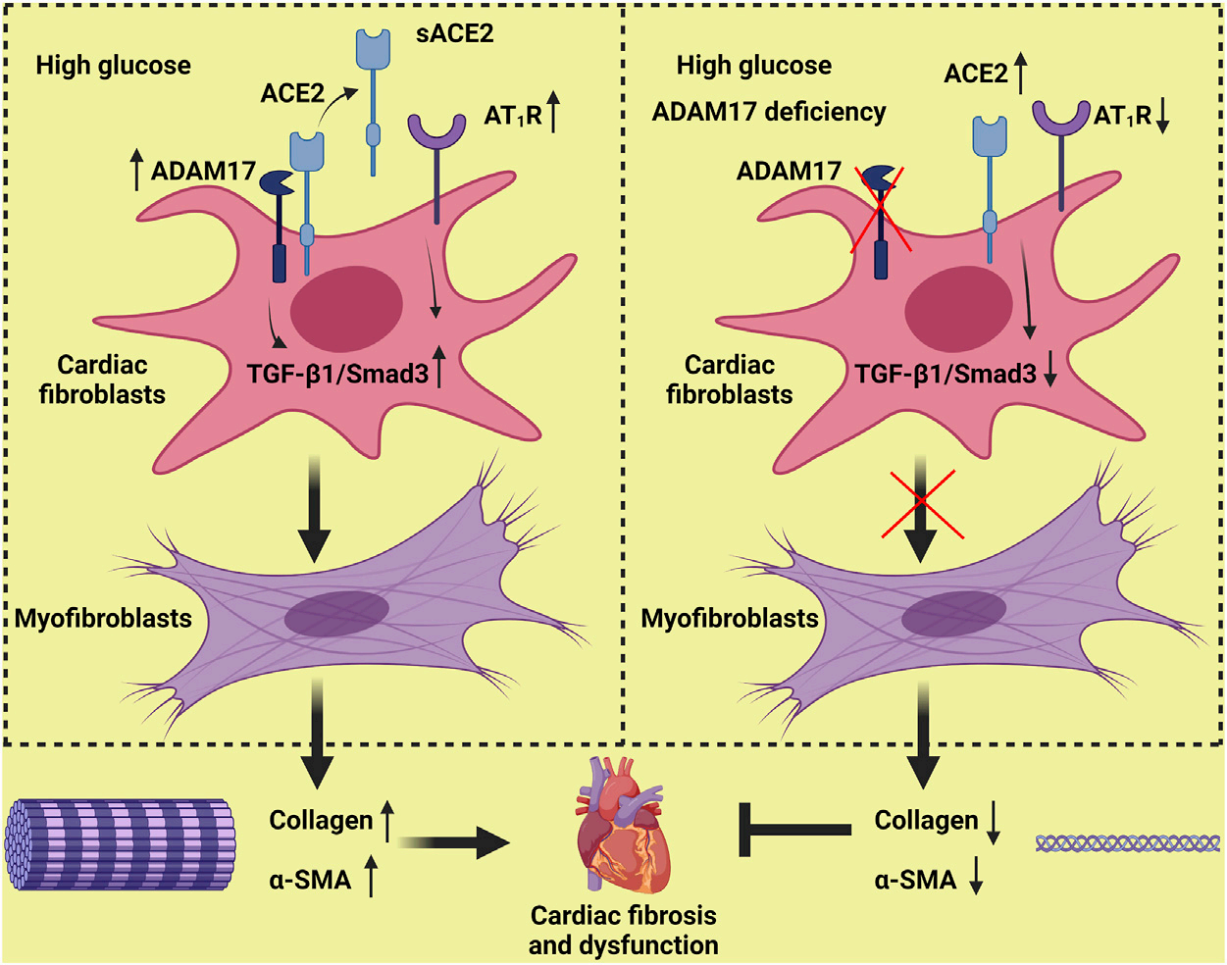
Proposed mechanisms underlying the roles of ADAM17 in cardiac fibrosis. High glucose increases ADAM17 activity, ACE2 shedding and AT1R expression, which activates TGF-β1/Smad3 signaling in cardiac fibroblasts, leading to an enhanced myofibroblast transformation and collagen deposition in the myocardium. ADAM17 deficiency attenuates ACE2 shedding and AT1R expression, which inactivates TGF-β1/Smad3 signaling in cardiac fibroblasts, leading to a reduced myofibroblast transformation and collagen deposition in the myocardium, thus ameliorating diabetes-induced left ventricular dysfunction and cardiac fibrosis.

In conclusion, ADAM17 knockdown ameliorated while ADAM17 overexpression worsened cardiac dysfunction and cardiac fibrosis in diabetic mice. The mechanism may involve reduced ACE2 shedding and myofibroblast transformation through inhibition of TGF-β1/Smad3 pathway by ADAM17 knockdown (Figure 7). Targeting ADAM17 may provide a promising approach to prevention and treatment of cardiac fibrosis in DCM.

## Data Availability

The original contributions presented in the study are included in the article/Supplementary Material, further inquiries can be directed to the corresponding authors.
