# Supplementary figures and images for: ADAM17 knockdown mitigates while ADAM17 overexpression aggravates cardiac fibrosis and dysfunction via regulating ACE2 shedding and myofibroblast transformation
Source: Front Pharmacol. 2022 Oct 14;13:997916. doi: 10.3389/fphar.2022.997916 (PMC9613967; doi:10.3389/fphar.2022.997916)

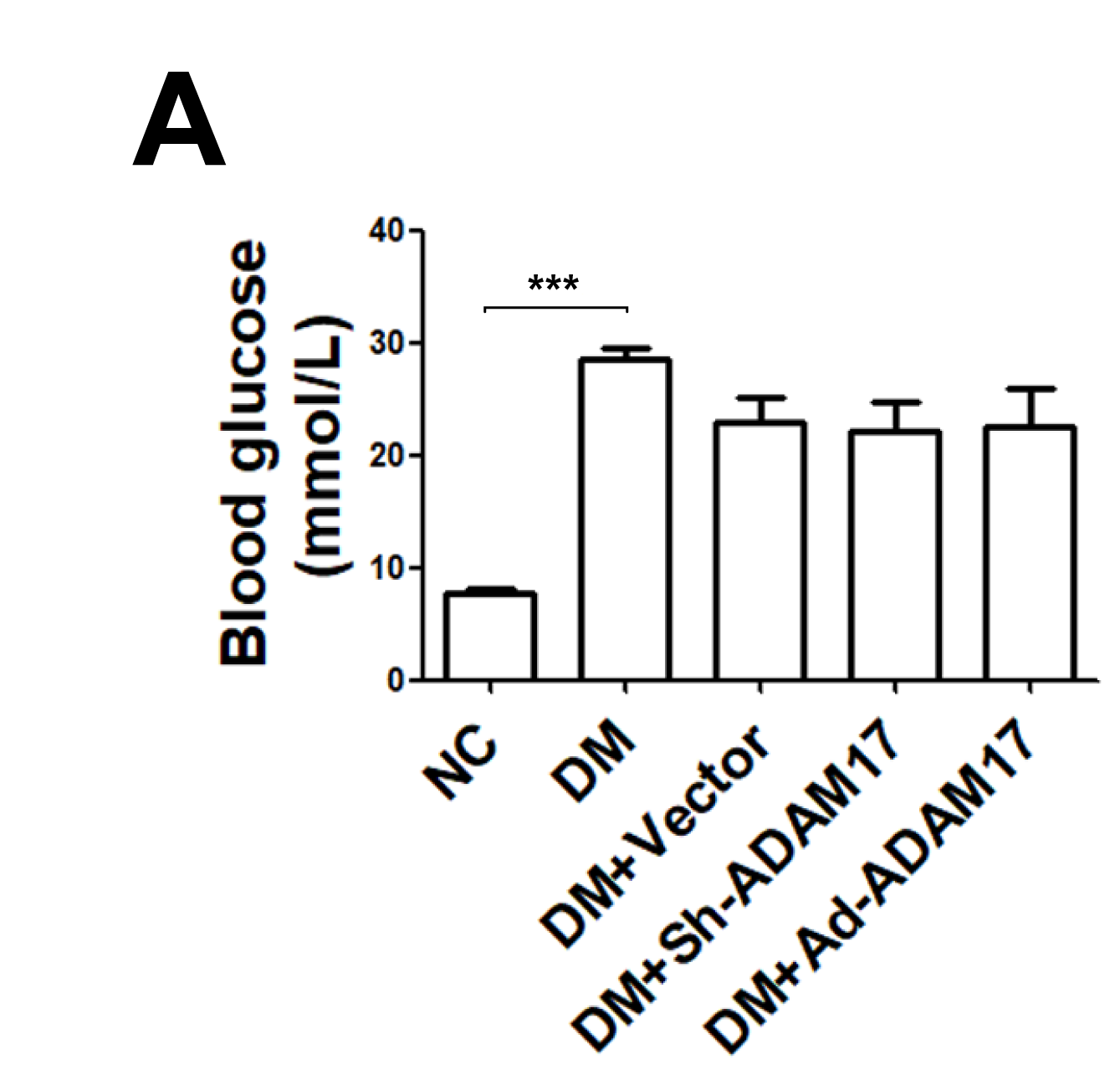

Supplement: Supplementary file 1 [file Image1.TIF]
