# Supplementary material for: ADAM17 knockdown mitigates while ADAM17 overexpression aggravates cardiac fibrosis and dysfunction via regulating ACE2 shedding and myofibroblast transformation
Source: Front Pharmacol. 2022 Oct 14;13:997916. doi: 10.3389/fphar.2022.997916 (PMC9613967; doi:10.3389/fphar.2022.997916)
Supplement: Supplementary file 2 [file DataSheet1.docx]

Supplementary Materials for

**ADAM17 knockdown mitigates while ADAM17 overexpression** **aggravates cardiac fibrosis** **and dysfunction via regulating ACE2 shedding and** **myofibroblast transformation**

***By:***

Jing Cheng ^1,2, a^, Fei Xue ^1, a^, Cheng Cheng ^1^, Wenhai Sui ^1^, Meng Zhang ^1^, Lei Qiao ^1^, Jing Ma ^1^, Xiaoping Ji ^1^, Wenqiang Chen ^1^, Xiao Yu ^3^, Bo Xi ^4^, Feng Xu ^5^, Guohai Su ^6^, Yuxia Zhao^1, 7^, Panpan Hao ^1,^ *, Yun Zhang ^1, 6^ *, Cheng Zhang ^1, 6^ *

***From:***

^1^ The Key Laboratory of Cardiovascular Remodeling and Function Research, Chinese Ministry of Education, Chinese National Health Commission and Chinese Academy of Medical Sciences, The State and Shandong Province Joint Key Laboratory of Translational Cardiovascular Medicine, Department of Cardiology, Qilu Hospital, Cheeloo College of Medicine, Shandong University, Jinan 250012, Shandong, China;

^2^ Heart Center and Beijing Key Laboratory of Hypertension, Beijing Chaoyang Hospital, Capital Medical University, Beijing 100020, China;

^3^ Key Laboratory Experimental Teratology of the Ministry of Education, Department of Physiology, School of Basic Medical Sciences, Cheeloo College of Medicine, Shandong University, Jinan, China;

^4^ Department of Epidemiology, School of Public Health, Cheeloo College of Medicine, Shandong University, Jinan, China;

^5^ Department of Emergency Medicine, Chest Pain Center, Shandong Provincial Clinical Research Center for Emergency and Critical Care Medicine, Qilu Hospital, Shandong University, Jinan, China;

^6^ Cardiovascular Disease Research Center of Shandong First Medical University, Central Hospital Affiliated to Shandong First Medical University, Jinan, China;

^7^ Deaprtment of Traditional Chinese Medicine, Qilu Hospital, Cheeloo College of Medicine, Shandong University, Jinan 250012, Shandong, China.

**^a^** These authors contributed equally to this study.

*** *Corresponding authors:*** Yun Zhang, email: [zhangyun@sdu.edu.cn](mailto:zhangyun@sdu.edu.cn), or Panpan Hao, email: [panda.how@sdu.edu.cn](mailto:panda.how@sdu.edu.cn), or Cheng Zhang, email: [zhangc@sdu.edu.cn](mailto:zhangc@sdu.edu.cn), Department of Cardiology, Shandong University Qilu Hospital, No. 107 Wen Hua Xi Road, Jinan 250012, Shandong, China

**Supplementary Table 1. Primers used for RT-PCR analysis**.

| Gene | Sequence 5’-3’ | Primers |
| --- | --- | --- |
| ADAM17 (Mus) | AGGACGTAATTGAGCGATTTTGG | Forward |
|  | TGTTATCTGCCAGAAACTTCCC | Reverse |
| ACE2 (Mus) | CAACCCAAAGAACCCACAAG | Forward |
|  | CTCTTCATACAACGGCCTCAG | Reverse |
| β-actin (Mus) | CACTGTGCCCATCTACGA | Forward |
|  | GTAGTCTGTCAGGTCCCG | Reverse |

**Supplementary Figure 1. (A)** Blood glucose levels in 5 groups of mice. Data were expressed as the means ± SEM. ****P* < 0.001. n=6 per group.
